# Design and Analysis of Particulate Matter Air-Microfluidic Grading Chip Based on MEMS

**DOI:** 10.3390/mi10080497

**Published:** 2019-07-26

**Authors:** Tingting Chen, Jianhai Sun, Tianjun Ma, Tong Li, Chang Liu, Xiaofeng Zhu, Ning Xue

**Affiliations:** 1 School of Electronic, Electrical, and Communication Engineering, University of Chinese Academy of Science (UCAS), Beijing 100190, China; 2 State key laboratory of transducer technology, Institute of Electronics, Chinese Academy of Science, Beijing 100190, China; 3 Beijing Municipal Institute of Labour Protection, Beijing 100054, China

**Keywords:** atmospheric particle size classification, air-microfluidic chip, virtual impactor, particulate matter

## Abstract

Atmospheric particulate matter (PM) air-microfluidic grading chip is the premise for realizing high-precision PM online monitoring. It can be used as an indispensable basis for identifying pollution sources and controlling inhalable harmful substances. In this paper, based on aerodynamic theory and COMSOL numerical analysis, a two-stage PM air-microfluidic grading chip with cut-off diameters of 10 μm and 2.5 μm was designed. The effects of chip inlet width (*W*), main flow width (*L*), second channel width (*S*), and split ratio (*Q*_1_/*Q*) on PM classification efficiency were analyzed, and optimized design parameters were achieved. The collection efficiency curves were plotted according to PM separation effects of the chip on various particle sizes (0.5–15 μm). The results indicate that the chip has good separation effect, which provides an efficient structural model for the PM micro-fluidization chip design.

## 1. Introduction

In recent years, with the rapid development of social industrialization and urbanization, problems such as automobile exhaust, industrial waste gas, construction dust, and garbage incineration are becoming increasingly serious, making the environment suffer from the most serious atmospheric particulate pollution ever [1]. High concentration of particulate matter (PM) has caused frequent haze, seriously affecting the air quality and population’s health [2]. The distribution of the PM particle size is from several nanometers to several hundred micrometers. The health harmful inhalation particles are within the above size ranges [3,4]. Among them, because of the small size, inhalable particulate matter (PM10) and fine particulate matter (PM2.5) can be deposited on the respiratory tract, alveoli, and so on [5,6]. Therefore, it is very essential to develop a low-cost and portable aerosol sensor for PM10 and PM2.5 monitoring.

When monitoring the PM concentration, people need to know the size information of the PM [7]. It is particularly significant to separate the PM size for a PM detecting system [8]. Grading sampling is typically based on the inertia of the particles to select them within a given range [9,10]. Widely used methods include impact particle size distribution sampling [11], centripetal particle size distribution sampling and cyclone separation sampling [12], where the centripetal particle size distribution sampling is also called “virtual impactor” [13,14]. Among these techniques, the particles are accelerated by the nozzle to obtain certain inertia. Particles that are smaller than the required cut-off diameter remain in the major flow, while the larger ones are separated by the wall or plate [8,9]. With the rapid development of automation and information technology, atmospheric particulate matter grading monitoring has been transferred from manual sampling to laboratory analysis, turning to miniaturization, intelligent and integrated on-site monitoring [10]. In 2014, I. Paprotny et al. [15,16] first proposed microfluidic technology, using particle size grading theory and numerical model analysis to design and manufacture a single-stage (PM2.5) separation device for PM monitor with small size and high sensitivity [17]. Subsequently, a number of PM air-microfluidic sensors have emerged [7,9,10,18,19]. The air-microfluidic technology, which, combined with micro-electro- mechanical system (MEMS), has the characteristics of high classification efficiency, simple structure, miniaturization and low cost. It has become the cutting-edge technology for particle size separation of atmospheric particles.

In this work, a microfluidic grading chip for atmospheric particulate matter with cut-off diameter of 10 μm and 2.5 μm was designed based on aerodynamic theory and COMSOL numerical analysis. We adopted COMSOL as a calculation tool, in which the separation trajectories of different sizes particles in the chip were accurately predicted using the finite element method. Furthermore, we studied the velocity fields and pressure fields of the virtual impactor (VI), the trajectories of the particles, and the collection efficiency. In order to optimize the chip structure, we mainly focused on the following four important parameters: inlet width (*W*), main flow through width (*L*), second channel width (*S*), and split ratio (*Q*_1_/*Q*). Through the experimental verification of the PM separation effect of various particle sizes (0.5–15 μm), the collection efficiency curve was fitted. The design of the air-microfluidic grading chip provided an efficient model [19]. In addition, we used a combination of 2D model and 3D model to make the numerical results more realistic.

## 2. Theoretical Analysis

When a particle flows around an obstacle, its trajectory is related to the size of the particle. Theoretically, the finest particle moves completely with the gas streamline, and the coarsest one moves in a straight line by its inertia nature. Most of the particles are between these two extremes [20].

**Figure 1:**
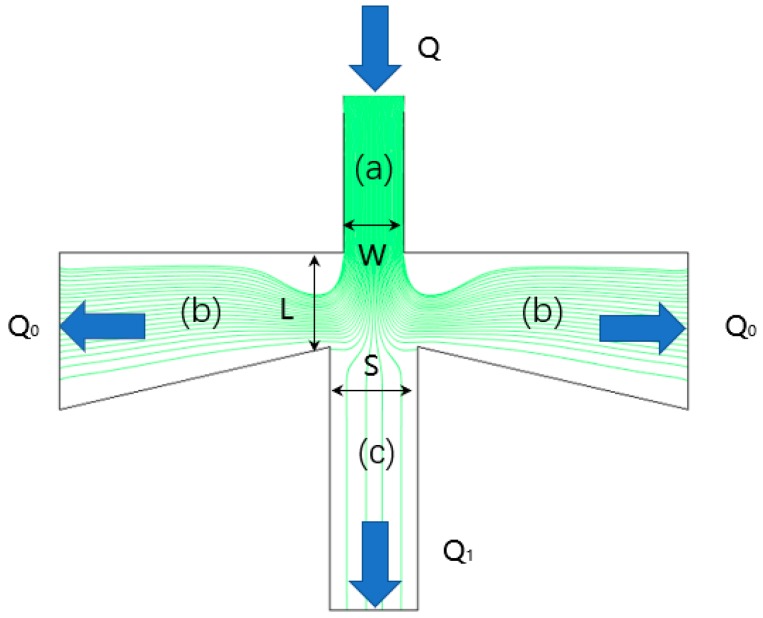
Streamline distribution of particulate matter (PM) with different particle sizes in virtual impactor (VI).

The two-stage air-microfluidic chip classifies the PM with different particle sizes by an inertial size separator, commonly referred to as a virtual impactor (VI). Figure 1 shows the streamline distribution of PM with different particle sizes in VI.

The VI can divide atmospheric particulate matter into two size-based categories via an accelerating jet (a). Most of the airflow flows through the main channel (b) due to the pressure at the outlet [8,18], whereas only a small portion of the airflow transfers to the minor flow channel (c), leading most of the gas flow lines to transition 90°. With sufficient inertia, the lager particles move linearly into the secondary circulation channel (c). In contrast, the smaller ones follow the flow the dominant flow lines into the main flow channel (b).

Atmospheric particle size classification model is generally designed based on Marple theory with two important parameters: Stokes number (Stk) and flow field Reynolds number (R) [21].

R is the ratio of the inertia to the viscous force of the fluid. It is a dimensionless number and can be expressed as

(1) $$R=\frac{\rho_{g}uW}{\eta }$$

where $\rho_{g}$ is the fluid density, u is the gas flow rate, W is the width at the inlet of the device, and η is the dynamic viscosity of the air medium. In this design, the gas movement in the circulation channel should be laminar, so the Reynolds number should be in the range of 500–3000.

Stk (a dimensionless number), defined as the ratio of particle relaxation time to fluid characteristic time, describes the behavior of suspended particles in a fluid. Generally, $Stk_{50}$ is used to characterize the efficiency of an inertial size separator, which represents the Stokes number when the collection efficiency is equal to 50%. For a rectangular jet, it is typically 0.59 [16]. The corresponding $d_{50}$ is the cut-off diameter of the separator. Considering the slip correction ($C_{C}$), it can be expressed as

(2) $$d_{50}=\sqrt{\frac{9\eta W^{2}D(Stk_{50})}{\rho QC_{C}}}$$

where, Q is the volumetric flow rate of the gas passing through the inlet, D is the depth of the inlet of the air-microfluidic device, ρ is the particle density. $C_{C}$ represents the Cunningham correction coefficient, which can be expressed as

(3) $$C_{C}=1+\frac{2A\lambda }{d}+\frac{2Q\lambda }{d}e^{-\frac{bd}{2\lambda }}$$

where, *A* = 1.234, *Q* = 0.413, *b* = 0.904, *λ* is the mean free path of air molecules that value is 6.95 × 10^−8^ m. Therefore, it can be reduced to

(4) $$C_{C}\approx \{\begin{array}{c} 1+2.52\frac{\lambda }{d},d>2\lambda \\ 1+3.29\frac{\lambda }{d},d<2\lambda \end{array}$$

## 3. Configuration and Methods

### 3.1. Configuration of the Channels and VIs

**Figure 2:**
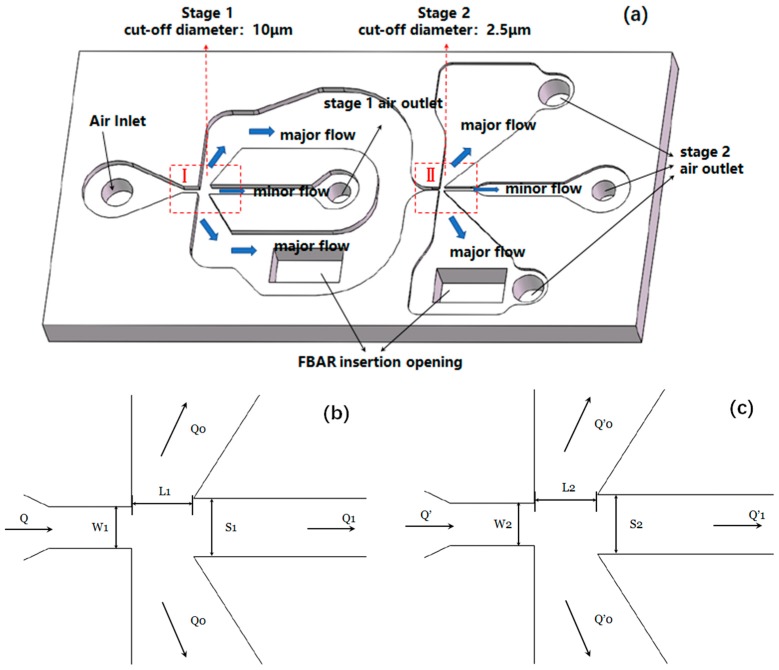
The configuration of the PM air-microfluidic grading channels and VIs (**a**). Partial enlargement of VI in PM microfluidic chip at I (**b**) and II (**c**), including the air inlet width and depth, the width of the main flow channel and the second channel, the volumetric flow of gas at the inlet and the split ratio.

In this work, the classification of PM with diameters of 2.5 μm and 10 μm is carried out. So, it is necessary to design the structures with two VIs (I, II in this case), connected in series. Figure 2 shows the configuration of the PM air-microfluidic grading channels and VIs.

Since the virtual impactor is a bottleneck in the airflow path, a certain pressure drop is generated when the air passes. Therefore, the design of the inertial size separator is optimized to minimize the pressure drop. Studies have shown that square nozzles have the lowest pressure loss [7]. Therefore, a square (*W* = *D*) nozzle section is chosen. Since the width of the second-stage particles separation device is smaller than the width of the first stage, the second stage (the cut-off diameter is equal to 2.5 μm) is selected as the standard, which is set to 200 μm, according to Equation (2).

The trajectory of PM in the microfluidic grading chip can be described as follows: When the PM follows the air entering the inlet, it firstly enters the separation of the first stage. The particles larger than 10 μm mainly enter the secondary circulation channel, followed by the carrier gas. As well as the outlet discharge, particles of less than 10 μm will follow the gas streamline into the main flow channel for the second stage of separation. Similarly, in the second stage separation, particles larger than 2.5 μm move substantially in a straight line and particles smaller than 2.5 μm enter the main circulation passage. With the above-mentioned method, the PM2.5 and PM10 particulates enter the respective mass sensing region, where a portion of the particles removed from the flow and deposited on the surface of the mass sensing film bulk acoustic resonator (FBAR) using a thermophoretic method.

### 3.2. Simulation Methods

We adopt COMSOL as a calculation tool, which is relatively well suited for the calculation of microsphere operations [22]. The simulation code of [18] is optimized, in which the separation trajectories of particles of different sizes in the chip are accurately predicted using the finite element method. Furthermore, we study the velocity fields and pressure fields of the VI, the trajectories of the particles, and the collection efficiency. The geometry of the VI can be simplified to two dimensions [7,16] during the optimization using finite element simulations performed by COMSOL. In addition, the 2D model can greatly reduce the number of calculations compared to the 3D model.

#### 3.2.1. Governing Equations

Laminar Flow (SPF) and Particle Tracing for Fluid Flow (FPT) modules in COMSOL were used in the design. Since the chip size and the Reynolds number are small, the movement of the gas in the separator is laminar, revealing in the air flow simulation by SPF.

In the 2D simulation, the Navier–Stokes equation [23] and the continuity equation are solved in order to calculate the flow and pressure of the incompressible liquid

(5) $$\rho \left(u_{flu}\cdot \nabla \right)u_{flu}=\nabla \cdot \left[-pI+\eta \left(\nabla u_{flu}+{\left(\nabla u_{flu}\right)}^{Τ}\right)\right]+F$$

(6) $$\rho \nabla \cdot \left(u_{flu}\right)=0$$

where, $\rho \left(u_{flu}\cdot \nabla \right)u_{flu}$ represents the unsteady inertia force, F is the volume force. ρ is the fluid density, P is the pressure, I is the unit diagonal matrix, η is the dynamic fluidic viscosity, and $u_{flu}$ represents the fluid velocity field.

FPT is used to simulate particles in the atmosphere, as the particles follow Newton’s second law

(7) $$\frac{d}{dt}\left(m_{p}v\right)=m_{p}F_{D}\left(u-v\right)$$

where u is the fluid velocity, $m_{p}$ is the mass of the pellet, v is the velocity of the pellet, and $F_{D}$ is the drag per unit mass. When the relative Reynolds number between the particle and the fluid is small, the drag per unit mass can be expressed as:

(8) $$F_{D}=\frac{18\eta }{\rho d^{2}}$$

To calculate the trajectory length of each particle, the following ordinary differential equations are solved for each particle

(9) $$\frac{d}{ds}\left(tl\right)=1$$

where tl is the length of the trajectory, s is the tangential direction of the particle motion at any given time.

#### 3.2.2. Boundary Conditions for Laminar Flow (SPF) and Particle Tracing for Fluid Flow (FPT)

In SPF boundary setting, the boundary conditions of the inlet and outlet are selected laminar flow. At the entrance and exit, fixed boundary conditions, also known as the Dirichlet boundary condition, are used to determine the value of the pressure.

(10) $$L_{entr,exit}\nabla_{t}\cdot \left[-pI+\eta \left(\nabla_{t}u_{flu}+{\left(\nabla_{t}u_{flu}\right)}^{Τ}\right)\right]=-p_{entr,exit}n$$

where, $L_{entr,exit},p_{entr,exit}$ represent the length and pressure of the entrance or exit, respectively, and n donates the outward normal bound. In FPT, a stream of particles over the first second for each inlet, with 30 particles per inlet, is defined. Defining three separate inlet features allows for improved visualization during results processing. When studying the trajectories of particles with different diameters in VIs, particles with one attribute can be added to each entry at each simulation.

#### 3.2.3. Optimization Method

The optimization approach is as follows: given the temporary size of the entire channel and targeted cut-off diameter (2.5 μm and 10 μm in this case), the total flow rate is calculated according to Equation (2). Then, the applied pressure and channel topology are adjusted to obtain a tangent point at the calculated air flow rate. According to the parameter optimization results of COMSOL simulation, Table 1 gives the optimized values of the characteristic size of the atmospheric particulate microfluidic chip. After calculation, the Reynolds number of the gas at the nozzle is in the range of 500–3000, which belongs to laminar flow.

**Figure 3:**
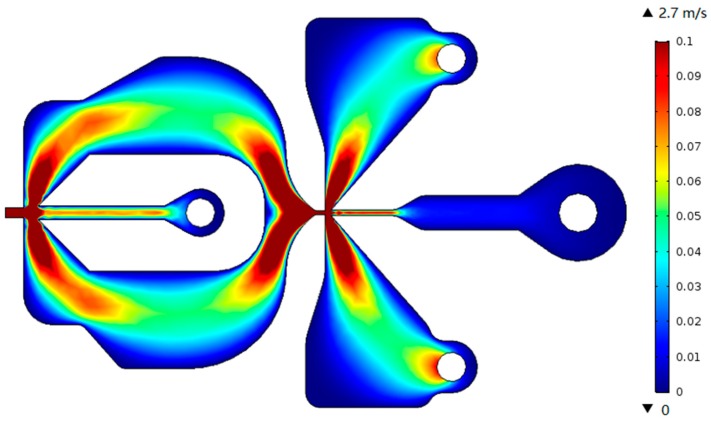
Flow field velocity distribution in PM air-microfluidic chip.

In numerical analysis, the flow field is calculated by the SPF interface, which ignores the force applied by the particles to the fluid. Two studies are designed: firstly, the flow field is solved in a study and then a separate study is implemented to calculate the trajectory of the particle based on the calculation of the flow field. The advantage of this solution is that when the particle size is changed to explore the collection efficiency characteristics of the model, it is not necessary to solve the flow field distribution again, so that it is not necessary to store a large number of time steps, saving the development cycle. The distribution of the flow field velocity in the PM air-microfluidic chip is shown in Figure 3.

## 4. Parameter Optimization and Numerical Analysis

The collection characteristics of the VI are determined by the relationship between the collection efficiency and the particle size. The ideal characteristic curve is a vertical line at the cutting point, which indicates that only particles smaller than the cutting particle diameter can follow the flow line into the main circulation, while the particles larger than that all move in a straight line into the second channel. However, the actual collection efficiency characteristic curve is an S-shaped curve. For the particle size separator, it is desirable that the collection efficiency curve has a higher steepness and is closer to the ideal characteristic curve for good separation effect.

In order to obtain an ideal collection efficiency curve, once the rectangular nozzle width W is determined, according to Equation (2), the sampling flow rate of the air inlet *Q* is determined. Thus, this section mainly studies the other three influencing factors: the second channel width *S*, the main flow passage width L and the split ratio (*Q*_1_/*Q*) on the collection efficiency curve of the inertia size separator.

### 4.1. Influence of Second Channel Width (S) and Main Flow Width (L) on Collection Efficiency

**Figure 4:**
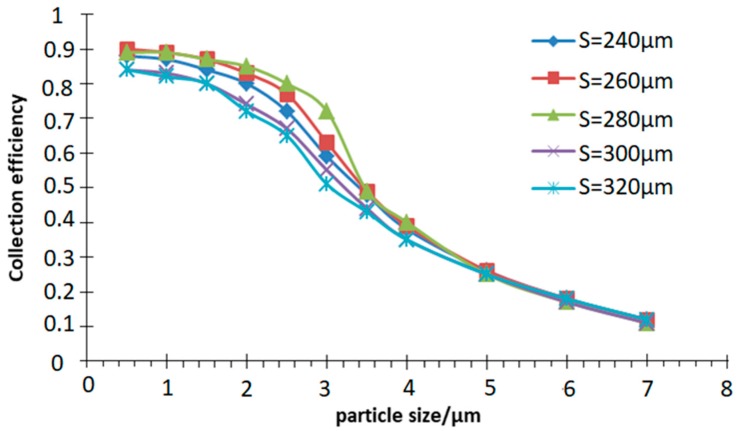
Collection efficiency curve with different second channel width (*S*).

**Figure 5:**
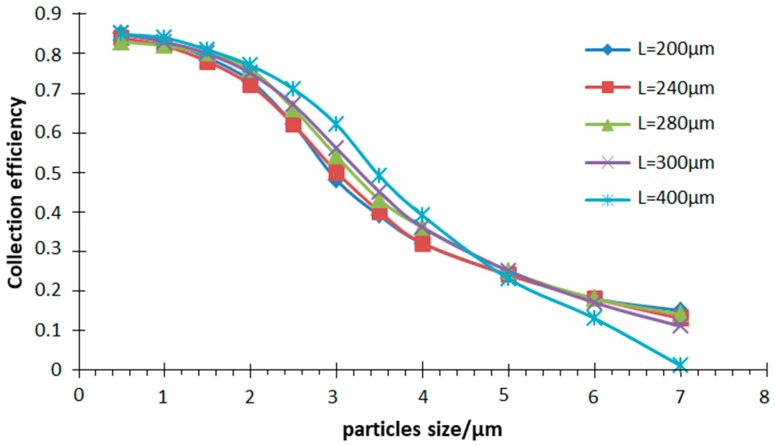
Collection efficiency curve with different main flow width (*L*).

*W* = 200 μm, *D* = 200 μm, *Q* = 6 mL/min, *Q*_1_/*Q* = 10% are used in the simulation. The width of the main flow channel and the second channel are varied respectively to obtain the collection efficiency curve in the simulation of the motion trajectory of atmospheric particles with different sizes, as shown in Figure 4 and Figure 5.

It can be seen from Figure 4 that the collection efficiency is greatly affected by the width *S* of the second channel within a certain range, and the influence thereof cannot be ignored when designing the inertia size separator. The results show that VI has the best separation effect when *S* is 280 μm, as the collection efficiency is the highest and the curve is the steepest. When the *S* is greater or less than 280 μm, the collection efficiency is reduced. At this time, the steepness of the curve becomes slow and the separation effect is deteriorated. Simultaneously, this value also meets the empirical value range, *S* is equal to 1.3–1.4 W.

**Figure 6:**
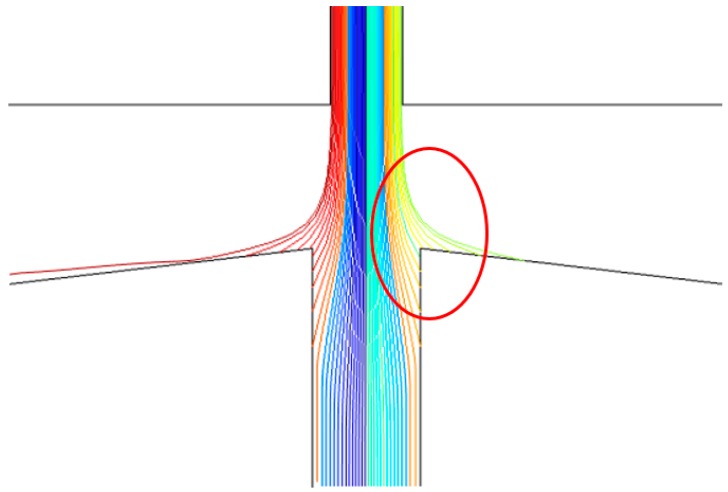
Movement of particles at *L* = 400 μm.

It can be seen from Figure 5 that within a certain range, the collection efficiency is less affected by the width *L* of the main flow channel than the width *S* of the secondary flow channel. When *L* changes in the range of 200–300 μm, the collection efficiency gradually increases and the steepness of the curve becomes steeper indicating that the separation effect of the inertial size separator gradually becomes better. When *L* is greater than 300 μm (such as *L* = 400 μm), although the collection efficiency is increasing and the steepness of the curve is steepening, but the inner wall loss of the particles is also becoming significant, as shown in Figure 6, which is not conducive to the collection of particles. Therefore, as *L* = 300 μm, the separation effect is good, and the value also conforms to the empirical value range, *L* is equal to 1.2–1.8 W.

### 4.2. Influence of Split Ratio (Q_1_/Q) on Collection Efficiency

When the inlet width and depth of the second stage are determined, according to Equation (2), the volumetric flow rate of the gas at the inlet can be obtained. In order to achieve concentration of atmospheric particulate matter, it is generally desirable to have the inertial size separator nozzle outlet in a state where the Reynolds number is high. However, a higher Reynolds number will increase the wall loss. To compensate for this loss, the split ratio (*Q*_1_/*Q*) is usually chosen to be no more than 0.1 [8].

**Figure 7:**
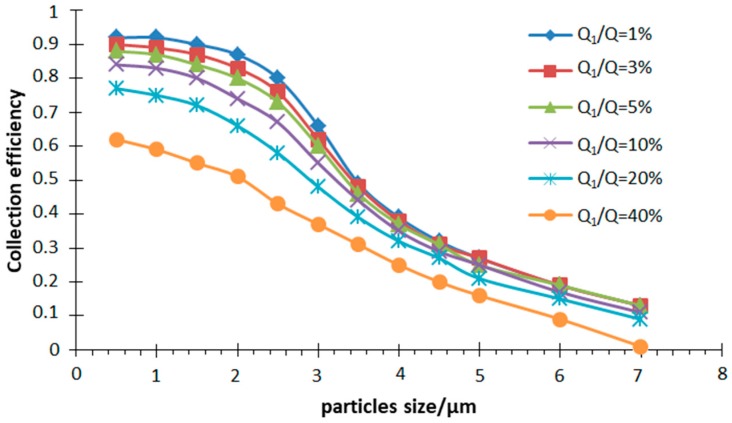
Collection efficiency curves at different split ratios (*Q*_1_/*Q*).

Having chosen *W* = 200 μm, *D* = 200 μm, *S* = 280 μm, *L* = 300 μm, *Q* = 6 mL/min, a different split ratio was simulated in the particle motion trajectory in the inertial size separator. The efficiency curve is shown in Figure 7.

It clearly indicates that the cut-off diameter decreases as the split ratio increases. It can be explained that the greater relative flow rate of the outlet of the secondary passage, the lower ability of the small-sized particles to change the motion state, and the greater the force of the airflow. In addition, for the particle size separator, the collection efficiency curve with a high steepness illustrates a good separation effect. It can be seen from the simulation results that when the split ratio is about 10%, the steepness of the collection efficiency curve is good. When the split ratio is decreased, the steepness of the collection efficiency curve does not change much, but the inner wall loss of the particles increased significantly. The large particle direction moves to increase the cut-off diameter of the inertial size separator. Conversely, the collection efficiency curve tends to be flat as the split ratio increases, which is not conducive to the separation of particles of different particle sizes. Therefore, when designing the inertial size separator, the split ratio is chosen to be 10%.

### 4.3. Analysis of Flow Rate and Pressure

**Figure 8:**
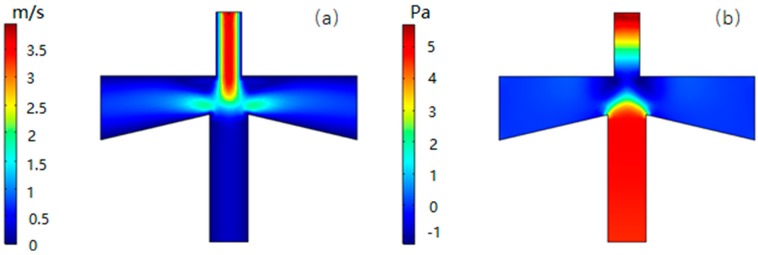
Velocity (**a**) and pressure (**b**) distribution in VI.

Figure 8a shows the flow velocity distribution at a volume flow rate of 6 mL/min. It can be seen that the fluid velocity distribution in the inertial size separator is axisymmetric, and the velocity of the gas in the main flow channel is higher than the velocity in the second channel. According to Equation (7), the larger the gas flow rate, the faster the velocity changes during the same time, and it is easier for the small particle size to follow the gas streamline motion. On the other hand, the trajectory of the suspended particles can be roughly estimated from the flow line of the gas, which is in good agreement with the expected result.

Figure 8b shows the pressure distribution corresponding to the flow velocity distribution. It can be seen that the pressure in the secondary circulation channel is significantly larger than the main circulation channel, defined as the differential pressure. The greater differential pressure can change the volumetric flow distribution of the gas at the inlet to ensure that the volumetric flow distribution of the primary and secondary flow channels is approximately 9:1. At the same time, the pressure drop of the inlet passage is small, about 6.6 Pa, which is also beneficial to the reduction of the power consumption.

### 4.4. Analysis of Trajectories with Different PM Size

**Figure 9:**
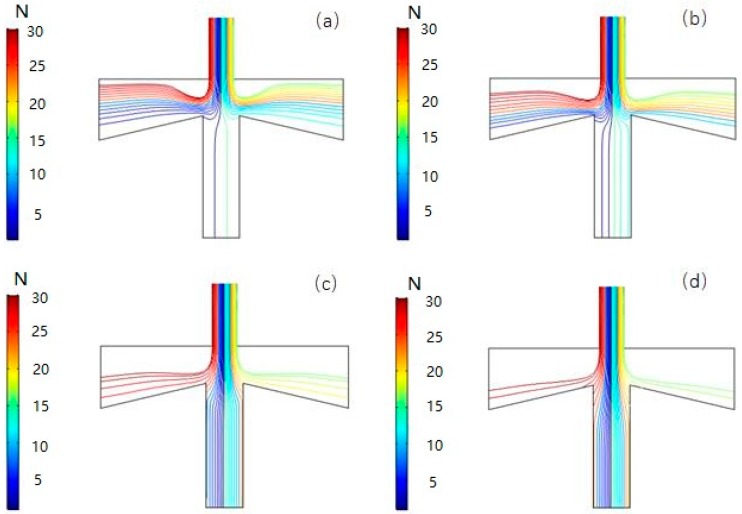
Particle motion trajectory of PM0.5 (**a**), PM2.5 (**b**), PM5 (**c**), and PM7 (**d**). In the figures, the trajectories of particles with different colors represent the number of each particle released at the inlet.

Figure 9 shows the simulation of the trajectories of particles with the four particle sizes: 0.5 μm, 2.5 μm, 5.0 μm, and 7.0 μm in VI using COMSOL 5.3a numerical analysis. Since the shape of the particles in the actual atmosphere is irregular, in order to facilitate the simulation, all the particles are assumed to be spherical, and the inlet volume flow rate is set to 6 mL/min, the split ratio is set to 10%. It can be seen from the results that fine particles (0.5 μm and 2.5 μm) tend to enter the main flow channel along the streamline while coarse particles (5.0 μm and 7.0 μm) are more likely to break away from the air flow line due to the larger inertia and move straight along the line into the second channel.

The above results show that the VI has a good separation effect for particles of different sizes, which lays a foundation for further design of atmospheric particulate microfluidic grading chips.

### 4.5. Analysis of Different Inlet Flows

**Figure 10:**
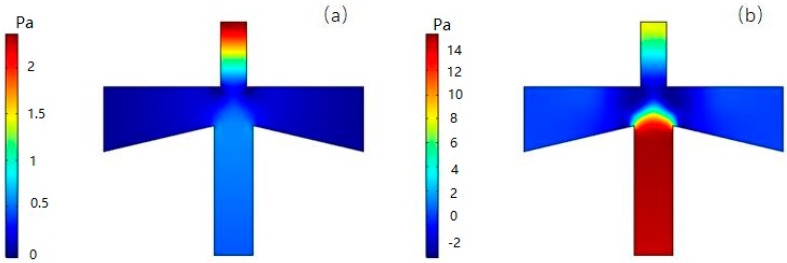
Pressure distribution at inlet flow rates of 2 mL/min (**a**) and 10 mL/min (**b**), respectively.

**Figure 11:**
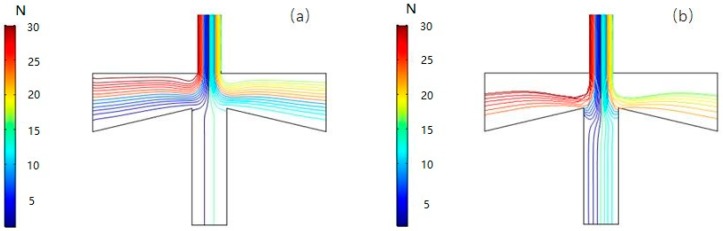
Particle trajectory of VI at 2 mL/min (**a**) and 10 mL/min (**b**).

Figure 10 and Figure 11 show the pressure distribution at a split ratio of 10%, the inlet gas volume flow rate of 2 mL/min and 10 mL/min, respectively, with the movement trajectory of particles (PM2.5) in the VI.

It can be seen from the simulation results that as the volumetric flow rate of the inlet gas becomes larger, the pressure difference between the main flow stage and the second channel also increases, and more gas is separated from the gas flow line and moves in a straight line to enter the secondary circulation channel, meaning that the cut-off diameter value is too large.

## 5. Result of the Simulation

**Figure 12:**
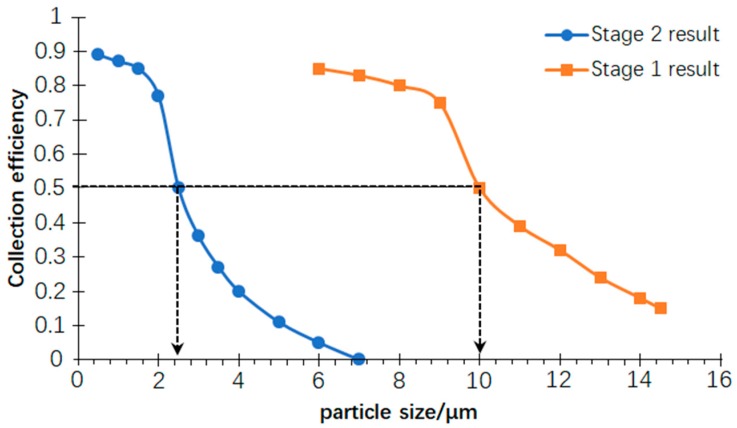
PM air-microfluidic grading chip collection efficiency curve.

A model of PM microfluidic grading chip was established, and atmospheric particles of different sizes were introduced from the inlet, respectively. The collection efficiency curve was obtained as shown in Figure 12. At this time, the volume flow rate of the inlet is 10.9 mL/min, which is larger than the theoretical calculation value, 6.9 mL/min.

As can be seen from the collection efficiency curve, the first stage inertial size separator has a cut-off diameter of 10 μm and the second one has a 2.5 μm cut-off diameter. Good steepness of both collection efficiency curves indicates that the PM air-microfluidic chip has a better separation effect when grading the particle size of the atmospheric particles.

## 6. Conclusions

In this work, a two-stage PM air-microfluidic grading chip was designed based on aerodynamic theory and some related research. Through the COMSOL numerical analysis, the influence of main structural parameters, such as inlet width (*W*), main flow width (*L*), second channel width (*S*), and split ratio (Q_1_/Q), on performance is optimized. The collection efficiency has been plotted in satisfactory curves. Moreover, comparison among other devices regarding the number of grades, the fitted cut-off diameter, and the steepness of the collection efficiency curve was conducted. The results are shown in Table 2. It can be seen that the cut-off diameter we simulated is comparable to the target one (2.5 μm and 10 μm AD), and the steepness is better. In addition, we used a combination of 2D model and 3D model to make the numerical results more realistic. When the chip enters the actual preparation process, it is very necessary to give priority to the package strength according to the pressure drop of the inlet stage. To ensure good sealing, the use of adhesive bonding by applying dispenser-printed [24] epoxy is a better choice. The epoxy can provide a seal around the perimeter of the microfluidic circuit, as well as around the flow channel. Furthermore, the sensor should be finally sealed with silicone around the edges of the bonded die to prevent leaks and increase the structural stability of the device, and the preparation process and performance of this chip will be reported in detail in subsequent papers.

## Figures and Tables

**Table 1:** Variables of the pm air-microfluidic chip design.

Variable	VALUE	Unit
*W*_1_	500	μm
*L*_1_	750	μm
*S*_1_	700	μm
*W*_2_	200	μm
*L*_2_	280	μm
S_2_	300	μm
*D*	200	μm
*Q*	6.9	mL/min
*Q*_1_/*Q*	10%	-
*Q’*_1_/*Q’*	10%	-

**Table 2:** Comparison with other microfluidic channels for PM flow dynamics analysis.

Property	Ours	I. Paprotny et al. [16]	Kim et al. [8]
Grading number	2	1	3
The target cut-off diameter	2.5 μm and 10 μm	2.5 μm	6 μm, 2.5 μm, and 200 nm
The fitted cut-off diameter	2.5 μm and 10 μm	2.5 μm	4.8 μm, 1.9 μm, and 135 nm
Curve steepness	Both stages are good	Good	As the number of stages increases, the steepness deteriorates.
